# Do Global Regulators Hold the Key to Production of Bacterial Secondary Metabolites?

**DOI:** 10.3390/antibiotics8040160

**Published:** 2019-09-23

**Authors:** Sudarshan Singh Thapa, Anne Grove

**Affiliations:** Department of Biological Sciences, Louisiana State University, Baton Rouge, LA 70803, USA

**Keywords:** antibiotics, biosynthetic gene clusters, *Burkholderia*, gene regulation, global transcriptional regulator, MftR, ScmR, secondary metabolites

## Abstract

The emergence of multiple antibiotic resistant bacteria has pushed the available pool of antibiotics to the brink. Bacterial secondary metabolites have long been a valuable resource in the development of antibiotics, and the genus *Burkholderia* has recently emerged as a source of novel compounds with antibacterial, antifungal, and anti-cancer activities. Genome mining has contributed to the identification of biosynthetic gene clusters, which encode enzymes that are responsible for synthesis of such secondary metabolites. Unfortunately, these large gene clusters generally remain silent or cryptic under normal laboratory settings, which creates a hurdle in identification and isolation of these compounds. Various strategies, such as changes in growth conditions and antibiotic stress, have been applied to elicit the expression of these cryptic gene clusters. Although a number of compounds have been isolated from different *Burkholderia* species, the mechanisms by which the corresponding gene clusters are regulated remain poorly understood. This review summarizes the activity of well characterized secondary metabolites from *Burkholderia* species and the role of local regulators in their synthesis, and it highlights recent evidence for the role of global regulators in controlling production of secondary metabolites. We suggest that targeting global regulators holds great promise for the awakening of cryptic gene clusters and for developing better strategies for discovery of novel antibiotics.

## 1. Introduction

A large number of antibiotics are in clinical use, and they target a variety of pathways that range from cell wall, protein, and DNA synthesis to folate and nucleotide metabolism [1]. Over the last decade, occurrences of multiple drug resistant bacteria have been on the rise, and the development of new effective methods or drugs to combat such strains has become critical [2]. Studies focused on the identification and isolation of novel natural products from microbes that are effective against pathogenic bacteria or possess other clinical significance, e.g., anti-fungal, anti-cancer, and immunosuppressant activities, have become a priority [3,4]. A considerable number of current pharmaceutical drugs have been directly derived from, or inspired by, bacterial natural products or secondary metabolites. These secondary metabolites, which are not essential for growth under normal conditions, are produced by bacteria in response to environmental stress or host interaction and provide them with a competitive advantage. Many bacterial species produce bioactive secondary metabolites, of which members of the genus *Streptomyces* and lately the genus *Burkholderia* are prominent sources. *Streptomyces* is the source of about 80% of the antibiotics that are produced today, which includes neomycin, kanamycin, vancomycin, streptomycin, tetracycline, and chloramphenicol [5,6]. Proteins that are essential for the production of these bioactive compounds are generally encoded by large cryptic gene clusters, which remain silent under normal laboratory conditions, a circumstance that creates a hurdle in discovery of novel compounds [7,8]. This review article focuses on such cryptic gene clusters in the genus *Burkholderia* and on potential mechanisms for eliciting their expression.

When originally coined in 1992, the genus *Burkholderia* comprised seven species [9]. At present, nearly a hundred validly named *Burkholderia* species exist [10]. These *Burkholderia* species occupy diverse ecological niches (free living, saprophytic, obligate endosymbionts, phytopathogens, opportunistic pathogens, or obligate parasites) and they include species that can serve as biocontrol and bioremediation agents as well as pathogens. Some *Burkholderia* species, mainly *B. pseudomallei*, *B. mallei,* and members of the *B. cepacia* complex (Bcc), have caught attention because of their pathogenicity [11]. Genome sequencing of *Burkholderia* spp., driven largely by a desire to understand virulence mechanisms, has led to the discovery of a large number of cryptic natural product biosynthetic gene clusters [12]. The regulatory mechanism of some of these cryptic gene clusters has been studied in detail, primarily in *B. thailandensis*. *B. thailandensis*, which is a relatively non-pathogenic strain, shares a large number of genes with other members of the *B. pseudomallei* complex (Bpc), some of which are involved in synthesis of bioactive compounds.

The *B. thailandensis* genome features ~22 natural product biosynthetic gene clusters [12,13]. Understanding how these gene clusters are regulated by local or global regulators (repressors or activators) and how different stress conditions (such as oxidative stress, osmolarity stress, phosphate starvation, and amino acid starvation) or inducing ligands can elicit gene expression is key to unlocking the potential of these cryptic gene clusters. Here, we focus on how the gene clusters that are responsible for encoding proteins involved in the synthesis of well-characterized bioactive compounds are regulated by local transcription factors dedicated to a specific gene cluster and how global regulators exert control over multiple gene clusters. Based on recent reports that a multitude of compounds are concurrently produced under conditions such as antibiotic stress and that the corresponding gene clusters are induced by inactivating a single transcription factor, we propose that such global regulators hold the key to the discovery of novel compounds.

## 2. Bioactive Secondary Metabolites

### 2.1. Malleilactone

**Figure 1:**
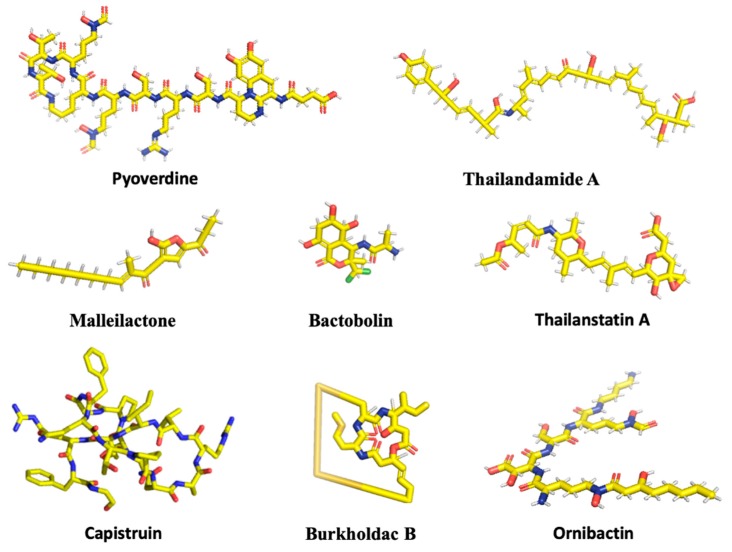
Chemical structures of selected secondary metabolites. Two-dimensional structures were obtained from PubChem (Malleilactone under the name Burkholderic Acid), except for Burkholdac B, which was obtained from ChEMBL (under the name Thailandepsin A) and Capistruin, for which the three-dimensional structure represents its conformation in complex with RNA polymerase (PDB ID 6N61). The complete amino acid sequence of Capistruin is **GTPGFQTPD**ARVISRFGFN, where bold letters denote residues linked by a backbone-sidechain lactam linkage to form the cyclic structure through which the C-terminal residues are threaded. Images were rendered with PyMol. C, yellow; O, red; N, blue; H, grey; S, orange; Cl, green.

Bacterially produced cytotoxic products are an important contributor to pathogenesis during host infection. To conserve energy, such compounds are only produced when needed and in response to inducing signals; they are, therefore, generally not synthesized under normal laboratory conditions, and this renders their identification and characterization particularly challenging. Malleilactone (also identified as Burkholderic acid [14]), is a polyketide synthase (PKS)-derived cytotoxic product, produced by species in the *B. pseudomallei* complex (Bpc), and it has received much attention, as it has been shown to be essential for *B. pseudomallei* and *B. thailandensis* to cause infection in *Caenorhabditis elegans* (Figure 1). Malleilactone can also inhibit growth of Gram-positive bacteria and it is cytotoxic to cultured mammalian cells [15,16,17]. Proteins that are encoded by the *mal* gene cluster produce it (Table 1).

**Figure 2:**
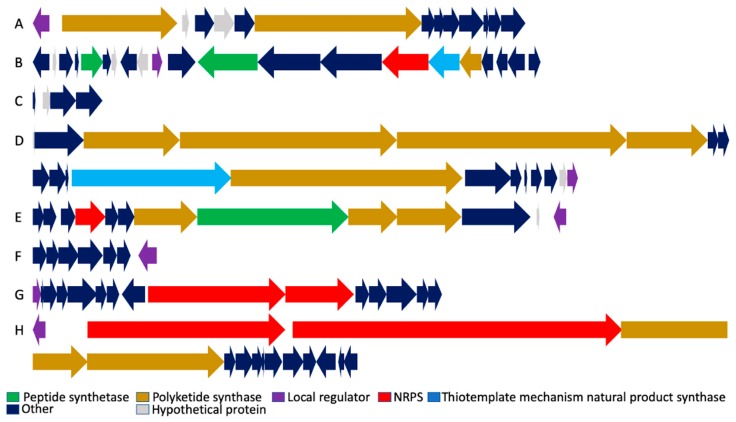
Organization of biosynthetic gene clusters. (**A**) Malleilactone. (**B**) Bactobolin. (**C**) Capistruin. (**D**) Thailandamide. (**E**) Burkholdacs. (**F**) Pyoverdine. (**G**) Ornibactin. (**H**) Thailanstatin. Genomic loci and individual gene annotations correspond to *B. thailandensis* E264 and are obtained from the Burkholderia Genome Database (https://www.burkholderia.com), except for the Ornibactin biosynthetic gene cluster (*B. cenocepacia* J2315; Burkholderia Genome Database [22]) and the Thailanstatin gene cluster (*B. thailandensis* MSMB43; NCBI GenBank JX307851.1).

In *B. thailandensis*, the *mal* cluster is an ~35 kb cryptic gene cluster with 13 open reading frames (BTH_II2088 to BTH_II2099). The *mal* cluster is highly conserved in *B. pseudomallei* and *B. mallei* with about 80–90% amino acid identity across these three species [14,15] (Figure 2A). The *mal* cluster has the same gene content in *B. pseudomallei, B. mallei,* and *B. thailandensis,* except that two annotated hypothetical genes that are upstream of *malA* and upstream of *malC* are absent in *B. thailandensis*. The *mal* cluster is divergent from the gene encoding the transcription factor MalR.

MalR has been shown to be essential for the expression of the *mal* cluster, and in both *B. thailandensis* and *B. pseudomallei*, MalR was shown to be required for the bacteria to infect *C. elegans* [18,19,20,21]. MalR is an orphan LuxR, which means that no cognate LuxI acyl homoserine lactone (AHL) synthase has been identified. The intergenic region between *malR* and *malA* contains a lux box-like region, to which MalR likely binds, and an intact lux box is required for the expression of *malA,* which indicates that MalR functions as an activator [13,15,18,19]. Generally, LuxRs respond to either endogenously and/or exogenously produced AHLs, but MalR does not respond to AHLs. However, the deletion of all three *luxI* genes in *B. pseudomallei* Bp82 led to ~10-fold greater production of Malleilactone, and expression was restored to wild type levels upon the addition of all three AHLs exogenously to the mutant culture. This led to the inference that AHL levels indirectly regulate the production of Malleilactone [19]. As outlined below, the AHL-mediated repression is likely achieved *via* the global regulator ScmR (Secondary Metabolite Regulator).

### 2.2. Bactobolin

Bactobolin, which is a polyketide-peptide (a C_6_-polyketide fused to a chlorinated hydroxy-valine residue), is produced by *B. thailandensis* (Figure 1). It was initially identified and characterized in *Pseudomonas* BMG13-A7 (1979 AD), where the compound was shown to have antibacterial as well as anti-tumor effects [23]. A number of studies have been conducted in *Burkholderia* spp. to understand the effect of the compound, its mechanism of action, and the genes that are involved in its synthesis. Approximately eight structurally different Bactobolin compounds have been identified in *B*. *thailandensis* (Bactobolin A-H) [24]. Bactobolins A-D have been tested for cytotoxicity to mammalian cells and antibacterial activity, with compounds A and C exhibiting more antibacterial activity than B and D. Further, there is a direct correlation between cytotoxicity and antibacterial activity [25]. The compounds seem to be particularly effective against Gram-positive bacteria. A mutational study in *B. subtilis* confirmed that Bactobolin targets the ribosome, but a different site than other known ribosome inhibitors [25]. Specifically, Bactobolin A’s biological activity derives from inhibiting protein synthesis by binding to a site in the 50S ribosomal subunit, which displaces tRNA from the P site of the ribosome [26].

Genes that are involved in synthesis of Bactobolin have been identified and characterized for *B. thailandensis* [25,27]. It is one of the few gene clusters that include both polyketide synthase (PKS)-and nonribosomal peptide synthetase (NRPS)-encoding genes (Figure 2B). An ~37 kb gene cluster, *btaA* to *btaU* (BTH_II1222-BTH_II1242), which includes the LuxI-LuxR system *btaI2* (BTH_II1227) and *btaR2* (BTH_II1231), is involved in the synthesis and regulation of Bactobolin (Table 1) [25,27]. The gene cluster is relatively conserved in *B. pseudomallei,* but not in *B. mallei*. The *btaI2-btaR2* quorum sensing pair is absent in *B. mallei* (most likely lost due to deletion of genes not required for virulence) [25,28]. The presence of the *btaI2-btaR2* system in the cluster suggests that the production of Bactobolin would be regulated in a quorum sensing-dependent manner, an inference that was confirmed by the generation of a quorum sensing-defective mutant [29]. That BtaI2 and BtaR2 control the production of Bactobolin has been established while using the *btaR2* mutant, which showed no antibiotic activity against Gram-positive bacteria. It is interesting to note that *btaR2* could also be regulated by the product of BtaI3 [27]. As discussed below, the expression of genes encoding BtaR2 and BtaI2, and therefore the *bta* gene cluster, is under the control of the global regulators ScmR and MftR (Major Facilitator Transport Regulator). Moreover, the production of Bactobolin has been suggested to be influenced by temperature, as its production was higher when *B. thailandensis* was grown at 30 °C as compared to 37 °C [25].

### 2.3. Capistruin

Capistruin belongs to the family of lasso peptides. Lasso peptides are bioactive peptides, which are ribosomally synthesized and post-translationally modified. They are characterized by an N-terminal macrolactam ring through which a C-terminal peptide tail is threaded [30,31,32]. The first lasso peptide, Anantin, was discovered in 1991 from a strain of *Streptomyces coerulescens* [33]. Capistruin from *B. thailandensis* was the first lasso peptide to be identified based on a genome-mining approach. This approach has since led to the more efficient identification of lasso peptides, and about 50 lasso peptides have been discovered so far. They have been classified into class I (which contains two disulfide bonds), class II (the most abundant type, which has no disulfide bonds, but the topology is held by steric hindrance), and classes III and IV (which both contain one disulfide bond that connects the ring to the tail or is within the tail region, respectively) [34]. Capistruin belongs to class II and it is similar to *E. coli* MccJ25 (Microcin J25; Figure 1).

The mature Capistruin is a 19 amino acid peptide (excised from a 47 amino acid precursor), in which the N-terminal 9 residues form the macrolactam ring through which the 10-residues long C-terminal tail is threaded [30,35,36]. Unlike other antibacterial compounds, Capistruin from *B. thailandensis* is effective against closely related *Burkholderia* and *Pseudomonas* strains [36]. This leads to the suggestion that Capistruin-producing *Burkholderia* species either encode an immunity protein or feature some modification of the target. Capistruin and Microcin J25 biological activity is due to their ability to inhibit RNA polymerase (RNAP), although for Microcin J25, the over-production of reactive oxygen species (ROS) through a possible secondary target has also been suggested [37,38,39]. Microcin J25 and Capistruin share the RNAP secondary channel as their binding site. Although Capistruin binds to *E. coli* RNAP as effectively as Microcin J25 in vitro, a nearly 10-fold higher concentration of Capistruin is required for inhibiting *E. coli* growth. Similarly, Microcin J25 can bind *Pseudomonas* RNAP as effectively as Capistruin, which inhibits *Pseudomonas* and *Burkholderia* growth, but no such inhibitory effect was seen with Microcin J25 [35,36]. A recently discovered lasso peptide, named Citrocin, which was isolated from *Citrobacter pasteurii* and *Citrobacter braakii,* showed similar effect; the lasso peptide was nearly 100-fold more potent as an RNAP inhibitor compared to Microcin J25, but a higher concentration of Citrocin was required to inhibit *E. coli* growth when compared to Microcin J25 [40]. Despite having the same functional target, the effect of the compounds seems to be species-specific. The most likely reason for this species specificity is a variation in cellular uptake/export [35]. Another plausible cause could be a variation in how the compounds inhibit RNAP. The crystal structure of Microcin J25 and Capistruin in complex with RNAP has revealed that Microcin J25 binds within the active site of RNAP, which limits or prevents access to NTPs, and its inhibition of RNAP is partially competitive with respect to NTP binding. This is not the case for Capistruin, which binds further away from the active site, its inhibition is partially non-competitive with NTP binding, and it lowers the rate of phosphodiester bond formation by eight-fold. This suggests that the Capistruin-mediated inhibition of RNAP catalysis is primarily due to interference with the proper folding of the trigger loop, a mobile element within the RNAP catalytic subunit [39].

By comparison to the *E. coli* MccJ25 gene cluster (*mcjABCD*), a similar cluster was found in *B. thailandensis* and was determined to be the Capistruin biosynthetic gene cluster (*capABCD*; Figure 2C). *capABCD* encodes (i) the capistruin precursor protein CapA (BTH_I2437a), (ii) two modifying enzymes for converting the precursor to mature lasso peptide, a putative protease CapB (BTH_I2438) and asparagine synthase CapC (BTH_I2439), and (iii) an ABC transporter CapD involved in export/immunity (BTH_I2440). CapD is most likely involved in the export of Capistruin from the cell, thereby mediating both transport and detoxification. Overproduction of the CapD homolog McjD in *E. coli* was sufficient to establish resistance against Capistruin [41]. The 4.5 kb lasso peptide gene cluster is conserved in *B. pseudomallei*. 

The mechanism of expression of lasso peptide biosynthetic genes is poorly understood (Table 1). The *E. coli mcjABCD* was shown to be up-regulated under iron deficient conditions [42]. Similarly, Capistruin production in *Burkholderia* was upregulated when cells were grown in minimal media under heat stress (42 °C). Both cases can be related in terms of nutrient deficient conditions, but if nutrient deficiency were the main trigger for expression, one would expect Capistruin production to be higher in the stationary phase as compared to the exponential phase. However, unlike many other secondary metabolites with antibacterial activity, Capistruin can be detected in exponential phase, and its synthesis is arrested while transiting from late exponential to early stationary phase [36], which suggests a different mode of regulation as compared to other biosynthetic gene clusters. While no local regulator has been reported, Capistruin biosynthesis genes are under the control of ScmR and MftR, as discussed below.

### 2.4. Thailandamide

Thailandamide, which is a linear, long-chain, unstable polyene natural product, is a fatty acid synthesis blocker (Figure 1). It is a broad-spectrum antibiotic that has effect against both Gram-positive and Gram-negative bacteria. The structure of three distinctive forms of Thailandamide have been elucidated (Thailandamide A, Thailandamide B, and Thailandamide lactone). Thailandamide A is effective against Gram-positives, but it is less effective against Gram-negative bacteria (except *Neisseria gonorrheae*). It is interesting to note that a change in the *E. coli* cell wall structure, which increased uptake, led to an increased efficiency of Thailandamide A. This prompted the suggestion that the otherwise broad-spectrum activity of Thailandamide is limited by poor uptake in Gram-negative bacteria [43]. A study involving insertional mutation for characterization of new antibiotics revealed Thailandamide B to be the major product formed by *B. thailandensis* [44]; this is contrary to other analyses, where Thailandamide A was shown to be the major product [43,45,46,47]. Thailandamide B was revealed to have bactericidal activity and it was shown to be toxic to human cells as well. Since Thailandamide is unstable, these differences might derive from variations in the extraction techniques; alternatively, mutations in genes encoding regulatory factors could lead to variations in the types of Thailandamide produced. For example, Thailandamide lactone, which was only detected in cells in which the *tha* gene cluster was highly active, displayed moderate anti-proliferative activity against tumor cell lines [46].

*B. subtilis* is sensitive to Thailandamide, and it was shown that a mutation in the *accA* gene, which encodes acetyl-CoA carboxylase, conferred resistance against Thailandamide A. Similarly, a single base mutation in *accA* was found in Thailandamide B-resistant *Salmonella* mutants. Further, in *B. subtilis*, over-expression of mutant AccA was sufficient to relieve the Thailandamide-induced inhibition. Thus, the cellular target for Thailandamide was suggested to be AccA protein or the AccA/AccD complex, which is involved in the first committed step of fatty-acid biosynthesis [43,44]. 

The *B. thailandensis* gene locus BTH_II1662-1681 encodes proteins that are involved in the synthesis of Thailandamide (BTH_II1662-1676), resistance (BTH_II1679), and regulation of its production (BTH_II1681/ThaA; Figure 2D). *thaC* or *accA*-2 is responsible for resistance against Thailandamide (*B. thailandensis* and closely related species are resistant to Thailandamide). The presence of a second copy of *accA* (*thaC*) likely affords the resistance to Thailandamide, which further strengthens the inference that AccA is the target for Thailandamide [43,44], as cells that express *thaC* are less susceptible to Thailandamide activity. It is interesting to note that several *Burkholderia* spp., which lack the *tha* gene cluster still encode a *thaC* homolog [43]. This could explain the Thailandamide resistance characteristic of these *Burkholderia* spp.

The mode of regulation of the *tha* biosynthetic gene cluster has not been elucidated or agreed upon completely (Table 1). Thailandamide production seems to be regulated by more than one gene product, and environmental cues appear to be responsible for its regulation. Under normal growth conditions, only vanishingly small amounts of Thailandamide are produced and exclusively in the early growth phase. The disruption of *thaA* (*luxR5*) abolished production of Thailandamide A, whereas the disruption of the *thaA* promoter resulted in increased production, likely on account of increased *thaA* expression. ThaA (LuxR5) has an AHL motif and is similar to LuxR regulators, but it has no known cognate LuxI [46]. In a study that was designed to elucidate a quorum sensing-controlled regulon in *B. thailandensis,* it was shown that ThaA is autoregulatory and represses *thaA.* Interestingly, *thaA* was activated upon addition of exogenous AHLs [48]. Whether ThaA directly responds to AHL levels or if *thaA* is activated by another transcription factor remains to be determined. Most likely, ThaA activates the expression of genes that are involved in Thailandamide synthesis. As cells progress from early growth phase, levels of AHLs increase, which should lead to an increased production of ThaA; therefore, the observed decrease in Thailandamide production speaks to the repression of the *tha* cluster by a different mechanism. As noted below, increased AHL-dependent production of ScmR might explain such repression. Recently, it has been shown that transposon-mediated disruption of *momS* (*BTH_I0633*) led to increased production of Thailandamide B. MomS has 66% sequence identity to AtsR (Adhesion and Type Six Secretion System Regulator), which has been shown to be a global regulator in *B. cenocepacia* [44].

### 2.5. Burkholdacs

Burkholdacs belong to the class of histone deacetylase (HDAC) inhibitors, which includes drugs, such as vorinostat, romidepsin, belinostat, and panobinostat (Figure 1). HDACs are a relatively new class of anti-cancer agents that induce death, apoptosis, and cell-cycle arrest. In eukaryotic cells, expression of genes is regulated through chromatin remodeling. One of the mechanisms by which such remodeling can be brought about is through either acetylation or deacetylation of lysine residues in core histones. While HATs (histone acetyl transferases), as the name suggests, acetylate the core histones, leading to uncoiled or less compact DNA, providing access to the transcription machinery, HDACs remove acetyl groups leading to the condensation of DNA around histone and repression of transcription. Thus, HDAC inhibitors prevent deacetylation, leading to an accumulation of hyperacetylated nucleosomes and differential gene expression [49].

Burkholdacs A and B from *B. thailandensis* were first isolated based on the over-expression of transcription factors linked to genes encoding secondary metabolite biosynthetic enzymes. In *B. thailandensis,* the *bhc* gene cluster includes two adjacent operons BTH_I2357-2358 and BTH-2359-2367, a hybrid-NRPS/PKS biosynthetic gene cluster (Figure 2E). The gene cluster has been shown to be under control of BTH_I2369 (encoded by *bhcM*), an AraC family transcription factor (Table 1) [50]. Members of the AraC/XylS family have three common regulatory functions: carbon metabolism, stress response, and pathogenesis [51]. Most members function as transcriptional activators, but some act as repressors or both, depending upon promoter architecture or the presence or absence of effectors [52,53].

The above-mentioned study involving an overexpression of transcription factors within or adjacent to NRPS/PKS gene clusters identified BhcM as an activator of the *bhc* gene cluster, but it did not reveal how *bhcM* expression is controlled. [50]. Members of AraC/Xyls can be classified into two groups: Either the signal receptor and regulatory function resides in same polypeptide or transcription of the regulatory protein is controlled by another regulator, be it repressor or activator [51]. BhcM seems to fall in the latter group, as discussed below.

### 2.6. Pyoverdines

Pyoverdines, fluorescent yellowish-green pigments, are the primary siderophores in *P. aeruginosa* (Figure 1). Siderophores are small, metal-chelating molecules with high affinity for Fe (III), which are produced by almost all bacteria and generally under iron limiting conditions. The role of siderophores becomes even more critical for pathogens that face a challenging low iron environment inside a host [54,55]. Besides acting as iron chelators, siderophores (catecholate types) can serve an anti-oxidant role during host-pathogen interactions [56]. Siderophores can also bind other essential metals, such as Mn, Mo, Co, and Ni, and deliver them to the microbe [57]. Various strains of *Pseudomonas* secrete different Pyoverdines, but commonalities in their structure include: (i) a fluorescent chromophore that is quenched upon binding of Fe^3+^, (ii) a strain-specific peptide that interacts with Fe^3+^ by chelating it, and (iii) an acyl side-chain bound to the chromophore whose functionality has not been completely understood [58]. Pyoverdines have been shown to be involved in both acquisition of iron and as signaling molecules for production of virulence factors [59,60,61,62]. No systematic analyses of Pyoverdine function have been reported in *B. thailandensis*. However, a study was carried out to determine the interactions between different cystic fibrosis pathogens discovered that Pyoverdine produced by *P. aeruginosa* inhibited the growth of *B. cenocepacia* J2315 [63].

The *B. thailandensis* chromosome harbors a predicted Pyoverdine gene cluster (BTH_II0229-0234) that is similar to *P. aeruginosa pvcABCD* (PyoVerdine Chromophore) cluster, which encodes proteins needed to synthesize Pyoverdine [17,64]. In *B. thailandensis*, a convergently oriented LysR-type transcriptional regulator (LTTR; BTH_II0235) is encoded immediately downstream of the cluster (Figure 2F). Similarly, *P. aeruginosa pvcABCD* has a convergently oriented *ptxR* that encodes an activator PtxR, also an LTTR. *ptxR* has two promoter sites, of which one is regulated in an iron dependent manner, while the other promoter is iron-independent [65].

*pvcABCD* expression has been shown to be repressed by presence of iron and to be positively regulated by the alternate sigma factor PvdS and by the activator PtxR [65,66]. A *ptxR* mutant did not produce detectable Pyoverdine, even under iron deficient conditions, which suggests that PtxR is an essential activator for expression of *pvc*ABCD in *P. aeruginosa* [66]. PvdS synthesis is repressed by another transcription factor, called Fur (Ferric uptake regulator), which utilizes Fe^2+^ as a corepressor [67]. A low level of iron causes the dissociation of ferrous ion from Fur, leading to the derepression of *pvdS*; in turn, PvdS can possibly facilitate the expression of *ptxR*. *B. thailandensis* encodes PtxR and several extracytoplasmic function (ECF) sigma factors, whose roles have not been determined. A Fur homolog, BTH_I1206, is also present in *B. thailandensis*. Thus, the Pyoverdine gene cluster may be similarly regulated in *B. thailandensis* as in *P*. *aeruginosa* (Table 1).

### 2.7. Ornibactin

Ornibactin, which is a tetrapeptide siderophore with an L-ornithine-D-hydroxyaspartate-L-serine-L-ornithine backbone (Figure 1), was first identified in *B. cepacia* [68]. Various strains of *Burkholderia* produce Ornibactin, but it has been primarily characterized in members of the Bcc. Ornibactin is produced under iron deficient conditions and its expression is completely inhibited by the presence of more than 15 μM of ferric iron in the media [69]. The function of Ornibactin, besides being an iron-acquiring molecule, has been established by studies of its function in different strains of *Burkholderia*. In *B. cepacia,* Ornibactin was shown to be critical for establishing infection in a murine chronic respiratory infection model. Moreover, it was noted that Ornibactin was critical in adherence and colonization [55,70]. Evolution of the role of Ornibactin was highlighted by a study that was conducted in *B. contaminans* MS14, where the production of Ornibactin was shown to be critical for the production of an antibacterial compound, which is effective against a wide range of plant-pathogenic bacteria [71].

The Ornibactin gene cluster, *orbA* through *orbS*, has been described in *B. cenocepacia* J2315 (BCAL 1688-1702). The gene cluster includes a gene encoding an ECF sigma factor, *orbS* (*BCAL 1688*), whose product OrbS has a high degree of similarity to PvdS (Figure 2G). The Ornibactin gene cluster contains a promoter region, to which OrbS could possibly bind and thus activate the expression of the gene cluster. Further, no production of Ornibactin was detected in an *orbS* mutant, suggesting that OrbS is an essential activator (Table 1). The *orbS* promoter has a region to which Fur protein may bind and repress its expression under iron sufficiency [69]. Thus, regulation of Ornibactin synthesis by OrbS and Fur could work in similar fashion as described for Pyoverdine.

An interesting thing to note in the case of the Ornibactin gene cluster, which is present in a large number of *Burkholderia* species, is that it has conserved NRPS genes (*orbI* and *orbJ*), but contains diversity within the genes that are involved in initiation, transport, regulation, and modification, suggesting the possibility for differential roles and regulation across species, as exemplified by the antibacterial activity of the *B. contaminans* MS14-derived Ornibactin [71]. While Ornibactin has been characterized from members of the Bcc, members of the Bpc produce the related siderophore malleobactin, with the biosynthetic genes being organized and regulated in a similar fashion [72].

### 2.8. Thailanstatin

Thailanstatin belongs to the spliceostatin class of natural products, which inhibit the spliceosome. Four forms of the compound (Thailanstatin A-D; Figure 1) have been isolated and characterized from *B. thailandensis* MSMB43 [73,74,75]. Alternative splicing, which is carried out by spliceosomes, generates an abundance of protein variants, however, cancer cells exhibit increased splicing levels, mutations in the splicing machinery, and aberrant alternative splicing. Thus, compounds that belong to the spliceostatin class can serve as potent anti-cancer agents [73,76].

FR901464, the first natural product of the spliceostatin family, was identified in 1996 from *Pseudomonas* sp. No. 2663 (subsequently, 16S rRNA sequence analysis showed the correct phylogenetic classification to be *Burkholderia sp.* FERM BP-342117 [77,78]), and it was shown to have marked anti-tumor activity [79]. As the compound is chemically unstable, a more stable methylated derivative, Spliceostatin A, was produced. In vitro assays revealed that Thailanstatin A, which was shown to be the most potent, could inhibit pre-mRNA splicing as efficiently as FR901464; moreover, it possesses anti-proliferative activity and it is more chemically stable than FR901464 [73,74]. Overall, Thailanstatin A has been shown to be less toxic to normal human cells and effective against human cancer cells lines. Spliceostatin A and FR901464 target the splicing factor 3b (SF3b) subcomplex of the U2 small nuclear ribonucleoprotein particle of the spliceosome, leading to the inhibition of pre-mRNA splicing and causing pre-mRNA leakage to the cytoplasm [80].

A gene cluster with homology to the *fr9* gene cluster, which encodes proteins that are required for production of FR901464, was discovered in *B. thailandensis* MSMB43 and it was named the *tst* gene cluster (Figure 2H); this gene cluster is not conserved in closely related species such as *B. thailandensis* E264. The *tst* gene cluster is a 78.1 kb DNA region comprising 15 ORFs (*tstA* through *tstR*). *tstA*, which is divergently oriented to the rest of the gene cluster, encodes a LuxR type transcriptional factor with no cognate LuxI within the gene cluster (Table 1). TstA has been suggested to be involved in the regulation of the gene cluster [73]. This genomic locus and its arrangement is very similar to the *mal* gene cluster arrangement in *B. thailandensis* E264 suggesting that TstA could possibly serve as an activator of the gene cluster similar to MalR. Whether TstA responds to AHL levels or any other ligand has not been reported.

## 3. Global Regulators of Biosynthetic Gene Clusters

### 3.1. ScmR (Secondary Metabolite Regulator)

As discussed above, dedicated pathway-specific regulators control the expression of many biosynthetic gene clusters. However, efforts to elucidate mechanisms by which cryptic biosynthetic gene clusters might be activated have recently led to the discovery of global regulators with a role in controlling an array of biosynthetic gene clusters. One of these regulators is ScmR, an LTTR, which has been shown to be involved in the production of various secondary metabolites in *B. thailandensis* E264. ScmR is conserved in *B. thailandensis* (BTH_I1403) with orthologs in *B. pseudomallei* and *B. mallei*. The *scmR* promoter contains a lux box, and gene expression was shown to be about two-fold greater when the cells were grown to higher cell density [16,48,81]. Moreover, no activation of *scmR* was seen in a mutant strain that was deleted for all three *btaI* genes, and therefore deficient in AHL synthesis. Similarly, a *B. pseudomallei* Bp82 triple *btaI* mutant exhibited ~3-fold lower *scmR* transcript levels when compared to wild type. Interestingly, the *B. thailandensis* triple *btaI* mutant featured an increased production of cryptic secondary metabolites, as also seen in the Δ*scmR* strain, which suggests that ScmR—and therefore production of some secondary metabolites—is regulated by quorum sensing. Further, production of AHLs is drastically reduced in the Δ*scmR* strain, indicating reciprocal regulation of AHL synthesis by ScmR. In the *B. thailandensis ΔscmR* strain, 13 of the >20 predicted biosynthetic gene clusters in *B. thailandensis* were differentially regulated [16]. We will focus on the role of ScmR in regulation of some of the compounds that are discussed above.

**Figure 3:**
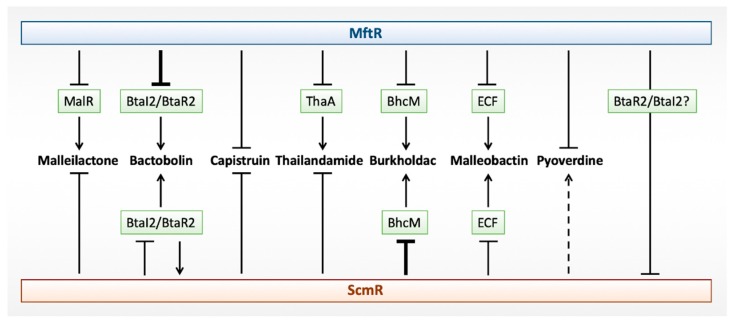
Regulation of biosynthetic gene clusters by Major Facilitator Transport Regulator (MftR) and Secondary Metabolite Regulator (ScmR). Cluster-specific regulators, where known, are identified in green. Repression is shown as lines, and activation as arrows. Deletion of *mftR* or *scmR* results in the greatest upregulation of Bactobolin and Burkholdac, respectively, as indicated by heavier lines. ScmR-mediated activation of Pyoverdine biosynthetic genes is marginal (dotted line). For ScmR-mediated regulation of the Bactobolin biosynthetic gene cluster, direct repression of *btaI2* was inferred, with BtaR2 reciprocally activating *scmR*.

The inactivation of *scmR* leads to a 7- to 13-fold upregulation of genes in the *bta* cluster, indicating that ScmR represses the production of Bactobolin [16]. The *bta* gene cluster is locally activated by BtaR2, which is induced by AHLs. AHLs are produced at a higher cell density and they serve to activate both BtaR2 and ScmR. The repression of genes in the *bta* cluster by ScmR could therefore be achieved by ScmR binding directly to the *btaI2* promoter and repressing transcription, consistent with the ~5-fold increased expression of *btaI2* in Δ*scmR*, forming a negative feedback loop that is designed to limit bactobolin production by attenuating activation by the AHL-dependent BtaR2 (Figure 3). Recently, a proteomic profiling of *B. thailandensis* during host infection revealed that ScmR is overexpressed in host-associated bacteria; while nine of 11 proteins encoded by genes that are repressed by ScmR were detected at lower levels, as expected, a Bactobolin biosynthetic enzyme was an exception. BtaC, encoded by *BTH_II1224*, was overproduced during infection. This suggests that, at least under infection conditions, Bactobolin synthesis is not solely determined through ScmR-mediated transcriptional repression [82].

The deletion of *B. thailandensis scmR* also revealed upregulation of genes involved in Capistruin biosynthesis, implicating ScmR as a repressor. During the stationary phase, AHL levels increase and *scmR* expression has been shown to be higher in presence of AHLs. Thus, the decrease in Capistruin production that was observed as cells enter stationary phase might be due to more efficient repression of Capistruin biosynthetic genes by ScmR [16]. Whether ScmR directly controls the Capistruin gene cluster or whether it controls the expression of a cluster-specific regulator remains to be determined.

The metabolomics analysis showed 61-fold increased production of Burkholdac A in Δ*scmR* cells, and the corresponding transcriptome analysis revealed 50- to 135-fold upregulation of the various genes in the *bhc* cluster [16]. Further, the complementation of the Δ*scmR* strain resulted in reduced production of Burkholdac, as seen in wild type cells, which indicated that ScmR represses the *bhc* gene cluster. Among the genes that were upregulated in Δ*scmR* cells is *BTH_I2369* (*bhcM*), encoding the AraC-family activator of the *bhc* gene cluster, BhcM. The repression of the *bhc* cluster by ScmR can therefore be explained by its repression of *bhcM*, possibly by direct binding to its promoter, resulting in failure to produce an essential activator. As AHLs are produced at higher cell density even under normal laboratory settings, which leads to increased production of ScmR and repression of *bhcM*, this would result in little to no production of Burkholdacs under these conditions.

The regulation of Malleilactone production by ScmR seems to be more complex. The metabolomics analysis of Δ*scmR* cells showed 210-fold overproduction of Malleilactone A, previously shown to be essential for the bacteria to cause infection in *C. elegans*. This overproduction was supported by the 8- to 18-fold upregulation of the different genes in the *mal* gene cluster and by the ~90% worm killing in a span of just 20 minutes when only ~5% of the worms were killed by wild type *B. thailandensis* [16]. Complementing the Δ*scmR* strain with plasmid-encoded *scmR* resulted in diminished production of Malleilactone, which verifies that ScmR acts as a repressor of Malleilactone synthesis. MalR has been established as an essential activator of the *mal* gene cluster, yet no change in expression of *malR* was noticed in the Δ*scmR* cells. This led the authors to the suggestion that ScmR possibly competes with MalR for binding to the *mal* promoter or that the accumulation of an unknown molecule only occurs in Δ*scmR* cells and functions as a MalR coinducer to activate the *mal* gene cluster further; we favor the first interpretation as no coinducers for MalR have been suggested in other studies. As discussed below, the global regulator MftR represses the *mal* gene cluster by also repressing the *malR* gene, which suggests an even more complex regulatory network. In a *B. pseudomallei* Bp82 Δ*scmR* strain, only ~4-fold increased production of Malleilactone was reported as compared to wild type cells, and the production was reduced to wild type levels upon complementing the mutant. In *B. pseudomallei* Bp82, *malR* expression was reported to modestly increase in a mutant deficient in AHL synthesis and *malR* transcript levels were two-fold higher in a Δ*scmR* strain, but only when cells were grown to late stationary phase and not in exponential or early stationary phase [16,19]. While the change in *malR* expression in the AHL mutant would be consistent with repression of *malR* by the quorum-sensing activated ScmR, the growth phase-dependent *malR* expression in Δ*scmR* cells points to regulation by a different mechanism.

### 3.2. Major Facilitator Transport Regulator (MftR)

*B. thailandensis* encodes 12 annotated MarR (Multiple Antibiotic Resistance Regulator) homologs, all of which are conserved in *B. mallei* and *B. pseudomallei* [83]. Members of the MarR family are transcription factors that are ubiquitous in the domains bacteria and archaea, and they have been shown to regulate various biological functions, such as response to environmental stress (for instance, antibiotic and oxidative stress or a change in pH), regulation of genes that are involved in virulence, and catabolism of aromatic compounds [84,85,86]. The genomic locus in which *mftR*, *BTH_I2391* in *B. thailandensis*, is divergently oriented to the *mftP-fenI* operon (*BTH_I2392* and *BTH_2393*) is conserved across members of the Bpc. MftR is a MarR homolog, while MftP (Major Facilitator Transport Protein) encodes an efflux pump and FenI is a predicted glycosyl hydrolase. MftR binds to the intergenic region between these divergently oriented genes, thereby repressing the expression of both *mftR* as well as the *mftP-fenI* operon [87].

MftR has been previously classified as a member of the MarR subfamily UrtR (Urate Responsive Transcriptional Regulator) [87,88]. Urate and xanthine, which are products of purine metabolism [8,89], bind MftR and attenuate its binding to DNA. Urate was predicted to bind MftR in a pocket that spans the DNA-binding and dimerization regions of the protein, a prediction that was based on the structure of the homologous urate-binding MarR protein HucR [87,90]. By binding in this pocket, the ligand is predicted to reconfigure the disposition of DNA recognition helices to create a conformation that is unfavorable for DNA binding. Urate is produced by host xanthine oxidase in response to bacterial infection at levels that can exceed 200 μM [91], which suggests that MftR might regulate genes upon host colonization. Indeed, determination of the MftR regulon by using an *mftR* knockout mutant revealed differential expression of ~400 genes, such as genes that are involved in biosynthetic processes, metabolism, and pathogenesis [17]. A total of 331 genes were upregulated, while 70 genes were down-regulated in the Δ*mftR* strain, which suggests that MftR is a negative regulator of most genes directly or indirectly under its control. Notably, a number of large biosynthetic gene clusters encoding various secondary metabolites, which are not expressed under normal laboratory settings were upregulated in the Δ*mftR* strain as well as when *B. thailandensis* was grown in media containing urate. As urate attenuates MftR binding to DNA, this observation is consistent with MftR functioning as a global repressor of these gene clusters, and it identifies urate as a common signal for the production of secondary metabolites. A proteomics analysis of differential protein accumulation in host-associated *B. thailandensis* showed a correlation between overproduced proteins and genes that are upregulated on the addition of urate, in accord with this interpretation [82]. The gene encoding ScmR is among those upregulated (2–3-fold) in the Δ*mftR* strain, whereas the expression of *mftR* is unaltered on the deletion of *scmR*, which indicates that MftR acts upstream of ScmR [16,17].

As discussed above, secondary metabolite production might be controlled by quorum sensing via AHL-activated expression of *scmR*. By contrast, MftR may be important for regulation of gene expression under conditions of host infection that involve the activation of host xanthine oxidase and therefore increased urate production. However, gene expression analyses indicate that these regulatory networks are intricately intertwined. The expression of genes that are involved in quorum sensing (*btaI2-btaR2, btaI3-btaR3*) as well as AHL levels are elevated in the Δ*mftR* strain, which directly implicates MftR in the quorum sensing circuit [17]. Thus, *scmR* expression might be repressed either by direct binding of MftR to the *scmR* promoter or—more likely—indirectly via the MftR-mediated regulation of AHL synthesis (Figure 3). Genes encoding Bactobolin, including *btaR2/btaI2*, are upregulated ~15-fold on the deletion of MftR or on the addition of urate, and *scmR* expression is modestly increased. This suggests that the ScmR-mediated repression of *btaI2* (and in turn the repression of the *bta* gene cluster) inferred to occur during balanced growth is bypassed when MftR is absent or the inducing ligand for MftR (urate) is present, and it is consistent with the overproduction of a Bactobolin biosynthetic enzyme in host-associated *B. thailandensis*, even under conditions of increased ScmR levels [82]. As *scmR* expression is increased in Δ*mftR* cells, a viable interpretation is that an activator of *btaI2* becomes abundant in the absence of MftR and competes with ScmR for binding; this activator could potentially be BtaR2, the expression of which is markedly increased in Δ*mftR* cells [17].

ScmR represses the expression of the local activator BhcM that is required for expression of the *bhc* gene cluster. Both *scmR*, *bhcM* and the Burkholdac biosynthetic gene cluster are upregulated ~two-fold in the Δ*mftR* strain, an expression pattern that suggests a more complex mechanism for the control of this gene cluster. The modestly increased expression of the Burkholdac biosynthetic genes in Δ*mftR* cells may therefore also derive from the accumulation of an activator of *bhcM* that can compete with ScmR for binding.

Expression of the *mal* and *tha* gene clusters that are involved in production of Malleilactone and Thailandamide, respectively, are upregulated two- to four-fold in *B. thailandensis* deleted for *mftR*. Genes encoding the local regulators MalR and ThaA are similarly upregulated in the Δ*mftR* strain, which indicates the possibility of direct regulation of these regulators by MftR. Both of these secondary metabolites and their respective gene clusters are upregulated in a Δ*scmR* strain, the *mal* gene cluster in particular, but no upregulation of the respective local regulators was seen. Increased expression of the *mal* gene cluster in Δ*mftR* cells could be explained by derepression of *malR*, which encodes an activator, while the regulation by ScmR might entail a competition between ScmR and MalR for the *mal* promoter, as noted above. A similar mode of regulation may pertain in the case of *thaA*, with ScmR more effectively competing with ThaA for the regulation of the *tha* gene cluster when *thaA* is repressed by MftR.

Several genes that were upregulated in *B. thailandensis ΔmftR* cells encode proteins that are involved in the production and transport of siderophores (Figure 3). This includes the gene clusters linked to production of Pyoverdine (BTH_II0229-0234) and the Ornibactin-like siderophore Malleobactin (BTH_I2414-2427). The mechanism of regulation of the Pyoverdine gene cluster remains unknown; specifically, we note that the convergent gene encoding a predicted LTTR activator of this gene cluster is not differentially expressed on the deletion of *mftR* [17]. The expression of genes in this gene cluster is modestly (<two-fold) lower in Δ*scmR* cells, which suggests moderate activation by ScmR. By contrast, the ECF sigma factor encoded as part of the Malleobactin gene cluster (BTH_I2427), which is predicted to activate expression based on comparison to the *P. aeruginosa* homolog PvdS, is upregulated in Δ*mftR* cells as well as in Δ*scmR* cells. Whether the gene encoding this sigma factor is directly or indirectly regulated by either MftR and/or ScmR has not been reported. This ECF is unlikely to participate in the activation of the Pyoverdine biosynthetic genes, as its upregulation in Δ*scmR* cells would have been expected to result in an increased expression of this gene cluster.

## 4. Trimethoprim as an Inducer of Cryptic Biosynthetic Gene Clusters

Genome mining has advanced the discovery of cryptic biosynthetic gene clusters and prompted efforts to optimize production of bioactive secondary metabolites. A variety of approaches, such as changes in fermentation conditions (media composition, temperature, and pH), co-cultivation, deletion of local regulators, and random insertional mutagenesis, have been successfully employed for the isolation and characterization of specific compounds in *Burkholderia* spp. For instance, production of Capistruin was greatly enhanced by growing *B. thailandensis* in M20 medium at 42 °C, while the isolation of Thailanstatin was optimized by growing cells in defined fermentation medium [35,36,73,74]. The production of siderophores was favored by growing the bacteria in iron deficient media or by cocultivation, and Malleilactone and Thailandamide were isolated as a result of an inducible promoter exchange strategy [15,44,71,92]. Such approaches have been inspired by the successful production of bioactive compounds from streptomycetes, which are well known as sources of clinically relevant compounds. In these species, strain development and metabolic engineering approaches have also been implemented for the improved production of select compounds [93]. While promising, metabolic engineering remains challenging, due to unintended consequences of competing pathways or the accumulation of toxic pathway intermediates.

In general, engineering approaches, including expression in heterologous hosts, have focused on the production of specific compounds. In contrast, chemical elicitors that create a stressful environment for the bacteria have the potential to induce the expression of multiple biosynthetic gene clusters. The activation of cryptic gene clusters by small molecules or ligands may not only increase the yield of individual compounds, but it is also a time and cost-saving technique by comparison to the more laborious culture optimization or systems metabolic engineering approaches [94,95]. Notably, a recent study that was focused on discovering novel elicitors of cryptic biosynthetic gene clusters in *B. thailandensis* found that, among the 640 candidates tested, a sub-lethal dosage of the antibiotic trimethoprim was the most potent elicitor [13]. It should be noted that a cocktail of trimethoprim and sulfamethoxazole (Co-trimoxazole) is a therapeutic drug prescribed for the treatment of *Burkholderia* infections [96,97]. Metabolomic profiling of cells that were treated with trimethoprim showed an upregulation of over 100 compounds, including molecules related to Capistruin, Malleilactone, Burkholdac, Thailandamide, and Bactobolin. Trimethoprim is a dihydrofolate reductase inhibitor, which prevents the conversion of dihydrofolate to tetrahydrofolate, a one-carbon donor that is essential in a variety of biosynthetic reactions, including the production of glycine, methionine, thymidine, and purines [98]. For trimethoprim to bind all of the structurally different, pathway-specific regulators of these cryptic biosynthetic gene clusters to induce gene expression would be unlikely. A more plausible scenario would be for trimethoprim or a metabolite that accumulates in cells treated with this antibiotic to modulate the function of a global regulator. Indeed, a correlation exists between the compounds that were detected on the addition of trimethoprim and the gene clusters upregulated when *B. thailandensis* is grown in media with urate added. However, for MftR, it has already been shown that trimethoprim does not directly bind to modulate DNA binding, and neither does 5-aminoimidazole-4-carboxamide ribonucleotide (AICAR), an intermediate in de novo purine biosynthesis shown to accumulate in the presence of trimethoprim [13,17]. Thus, the inducing ligand, which accumulates in cells grown with trimethoprim, has yet to be discovered.

## 5. Conclusions and Future Outlook

Secondary metabolites confer a competitive advantage to bacteria in a hostile environment, including the inhospitable environment that is created by host defenses, yet the mechanisms that regulate their production have not been completely deciphered. The gene clusters that encode enzymes responsible for the production of these secondary metabolites, whether being harmful virulence factors or novel compounds with potential clinical activity, such as antibiotics, generally remain silent or cryptic to conserve cellular resources. Understanding how the expression of these gene clusters is elicited could shed light on mechanisms of pathogenicity as well as advance the discovery of novel beneficial compounds. The identification of global regulators, which control cryptic biosynthetic gene clusters, has opened a possible path towards achieving these goals.

Global regulators have previously been shown to be involved in the regulation of specialized genes, such as those that are involved in pathogenesis, quorum sensing, and biofilm formation. Increased biofilm formation, which also aids in bacterial antibiotic resistance, is a characteristic of the Δ*scmR* strain. In a similar vein, increased anaerobiosis has been reported for the Δ*mftR* strain, a metabolic state that is critical for the survival of bacterial species in an oxygen-deprived environment, such as the interior of a biofilm and abscesses that are caused by infection. A role for both ScmR and MftR in virulence is further supported by their regulation of genes encoding enzymes that are responsible for the production of siderophores, which become critical in the iron-deficient environment inside a host [16,17,99,100]. These findings not only suggest that global regulators can control bacterial fitness in a host environment and increase their antibiotic resistance, but they also suggest global regulators as suitable targets for drugs. A recent study in *P. aeruginosa* showed that the global regulator MvfR (also known as PqsR), an LTTR, is involved in biofilm formation. A drug (M64) targeting MvfR interfered with biofilm formation of P. *aeugronoisa* and increased the antibiofilm activity of other antibiotics when used in conjunction with M64 [101]. 

The secondary metabolites that are discussed above likely represent just the tip of the iceberg and additional, novel secondary metabolites isolated from *Burkholderia* spp. await characterization. Inactivation of the global regulators ScmR and MftR is associated with remarkable changes in secondary metabolite production and a corresponding induction of biosynthetic gene clusters. Based on these observations, we predict that identifying global regulators in other bacterial species and understanding their regulatory mechanisms through a combination of genome-wide transcriptomics, metabolomics, and ChIP-Seq may enhance our chances of discovering potentially bioactive compounds as well as novel drug targets for pathogenic strains.

## Figures and Tables

**Table 1:** Local regulators of biosynthetic gene clusters.

Bioactive Compound	Gene Cluster	Local Regulator	Cellular Target/Function
Malleilactone	*mal*	MalR (Orphan LuxR)	Unknown
Bactobolin	*bta*	BtaR2 (LuxR)	50S Ribosomal Subunit
Capistruin	*cap*	Unknown	RNA Polymerase
Thailandamide	*tha*	ThaA (Orphan LuxR)	Acetyl-CoA Carboxylase
Burkholdacs	*bhc*	BhcM (AraC)	Histone Deacetylase
Pyoverdine	*pvc*	BTH_II2035 (LTTR)? Unknown	Siderophore
Ornibactin	*orb*	OrbS (ECF)	Siderophore
Thailanstatin	*tst*	TstA (Orphan LuxR)	Spliceosome
